# Production and effect of aldonic acids during enzymatic hydrolysis of lignocellulose at high dry matter content

**DOI:** 10.1186/1754-6834-5-26

**Published:** 2012-04-30

**Authors:** David Cannella, Chia-wen C Hsieh, Claus Felby, Henning Jørgensen

**Affiliations:** 1 Forest and landscape, University of Copenhagen, Rolighedsvej 23, Frederksberg C, DK-1958, Denmark

**Keywords:** GH61, High solids hydrolysis, Gluconic acid, Cellulose oxidation

## Abstract

**Background:**

The recent discovery of accessory proteins that boost cellulose hydrolysis has increased the economical and technical efficiency of processing cellulose to bioethanol. Oxidative enzymes (e.g. GH61) present in new commercial enzyme preparations have shown to increase cellulose conversion yields. When using pure cellulose substrates it has been determined that both oxidized and unoxidized cellodextrin products are formed. We report the effect of oxidative activity in a commercial enzyme mix (Cellic CTec2) upon overall hydrolysis, formation of oxidized products and impact on β-glucosidase activity. The experiments were done at high solids loadings using a lignocellulosic substrate simulating commercially relevant conditions.

**Results:**

The Cellic CTec2 contained oxidative enzymes which produce gluconic acid from lignocellulose. Both gluconic and cellobionic acid were produced during hydrolysis of pretreated wheat straw at 30% WIS. Up to 4% of released glucose was oxidized into gluconic acid using Cellic CTec2, whereas no oxidized products were detected when using an earlier cellulase preparation Celluclast/Novozym188. However, the cellulose conversion yield was 25% lower using Celluclast/Novozym188 compared to Cellic CTec2. Despite the advantage of the oxidative enzymes, it was shown that aldonic acids could be problematic to the hydrolytic enzymes. Hydrolysis experiments revealed that cellobionic acid was hydrolyzed by β-glucosidase at a rate almost 10-fold lower than for cellobiose, and the formed gluconic acid was an inhibitor of the β-glucosidase.

Interestingly, the level of gluconic acid varied significantly with temperature. At 50°C (SHF conditions) 35% less gluconic acid was produced compared to at 33°C (SSF conditions). We also found that in the presence of lignin, no reducing agent was needed for the function of the oxidative enzymes.

**Conclusions:**

The presence of oxidative enzymes in Cellic CTec2 led to the formation of cellobionic and gluconic acid during hydrolysis of pretreated wheat straw and filter paper. Gluconic acid was a stronger inhibitor of β-glucosidase than glucose. The formation of oxidized products decreased as the hydrolysis temperature was increased from 33° to 50°C. Despite end-product inhibition, the oxidative cleavage of the cellulose chains has a synergistic effect upon the overall hydrolysis of cellulose as the sugar yield increased compared to using an enzyme preparation without oxidative activity.

## Background

The use of renewable resources for the production of fuels and chemicals has been a continuing topic of interest. The bioconversion process involves the use of enzymes to convert cellulose into fermentable sugars, which are then substrate for further processing into mainly ethanol. The core pool of enzymes, known generally as cellulases, makes up a well established system of action divided in two groups: cellobiohydrolases and endoglucanases, plus a third component known as the β-glucosidase [1].

Despite significant progress in this field, the enzymatic deconstruction of the lignocellulosic biomass is not yet fully understood, especially regarding the action of non-hydrolytic enzymatic activities [2]. Cellulose-degrading microorganisms also produce accessory proteins that are co-regulated and co-expressed with the cellulase enzymes. These auxiliary proteins do not hydrolyze cellulosic material *per se*, but play a significant role in enhancing the yield by increasing the access of cellulases to the substrate and opening the crystalline structure: such enzymes are the swollenins and expansins [3]. A novel auxiliary enzyme activity capable of an oxidative cleavage of the glycosidic bond is currently classified in the Glycoside Hydrolase family 61 (GH61) [4]. Since Vaaje-Kolstad *et al.*[5] identified the oxidative process as a result of enzymatic activity, a variety of GH61-like proteins from different fungi as well as bacteria (GH61D from *P. chrysosporium*[6], GH61A from *T. auranticus*[7,8], several from *N. crassa*[9] and CelS2 (CBM33) from *S. coelicolor*[10]) have been isolated and studied. Notwithstanding the key role of GH61, the correct placing on the lignocellulosic degradation scenario, especially in relation to the classical cellulases, still remains ambiguous. Although a final model mechanism of action has not yet been found, some common features can be generalized: i) GH61s are metallo-enzymes that need a bivalent metal ion to act, and copper seems to be the metal ion coordinated in the active site; ii) since the oxidation of the glycosidic bond is the main activity, all GH61s need a reductant cofactor that works as an external electron donor: gallate, ascorbate, and the enzyme CDH (often up regulated and expressed together with GH61s [8]) are indicated to enhance the GH61s activity; iii) as substrate, aggregated cellulose is preferred: no activity was detected on soluble cellodextrines; iv) finally but most important, mass spectrometry and HPAEC analysis of reaction products of GH61s show a variety (different DP) of native as well as oxidized cellodextrines as a result of the glycosidic bond cleavage. Even though the oxidation may take place at several carbons in the glucose ring structure (C1, C4 or C6), the C1 oxidized (aldonic) cellodextrines are the most represented [5-10].

**Figure 1:**
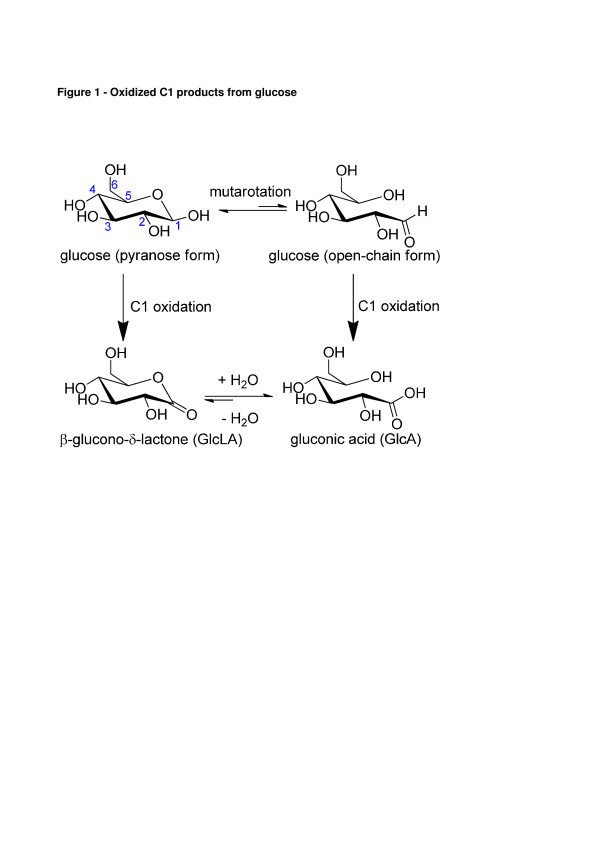
Oxidized C1 products from glucose.

During enzymatic deconstruction of lignocelluloses, the presence of exocellulase and β-glucosidase enzymes rapidly degrade native as well as oxidized cellodextrines into di- and monosaccharides. We suppose that cellobiohydrolases also hydrolyze those cellodextrines carrying a C1 oxidized glucose thereby releasing cellobiose and cellobionic acid as products. Similarly, we suppose that cellobionic acid is also one of the substrates for β-glucosidase. Thus the final products expected in a hydrolysate are native glucose and intermediate cellobiose, as well as their oxidized forms glucono-δ-lactone/gluconic acid and cellobio-δ-lactone/cellobionic acid respectively as shown in Figure 1. Once C1 oxidized cello-oligosaccharides are produced in solution, there exist a chemical equilibrium between their lactone and aldonic acid forms which is dependent on pH, temperature, and concentration [11]. The lactone form can hydrolyze non-enzymatically to the aldonic acid form. The rate of aldonic acid formation can be increased by lowering the pH, but lactonization as well as its reverse reaction does not alter the acidity of the solution [12]. During the enzymatic hydrolysis process at a pH of about 5, equilibrium tends to shift toward the aldonic acids. The presence of gluconic acid is relevant from an industrial bioconversion point of view because it has been proven to be a β-glucosidase inhibitor [13] and is also a non-fermentable sugar for *S. cerevisiae*[14], which in turn means that part of the potential glucose is lost as gluconic acid, that cannot be fermented into ethanol.

The boosting and synergetic effect of oxidative enzymes such as GH61 on lignocellulosic hydrolysis is well recognized [5,15] and oxidative enzymes are now present in commercially available cellulase preparations to improve the conversion yields [16]. An example is Novozymes Cellic CTec2 (used in this work) as opposed to its predecessor, the combined Celluclast 1.5 L/Novozym 188 mixture (Novozymes A/S, Bagsværd, Denmark). Despite the presence of two genes encoding for the GH61 family enzymes in *Trichoderma reesei*[17,18], oxidized products are generally not found in cellulose and lignocellulosic hydrolysate using commercial *T. reesei* cellulolytic systems [19].

Most of the papers cited above show the production of oxidized products by GH61 activity under ideal conditions using pure cellulose or PASC (phosphoric acid swollen cellulose) as substrate at low dry matter concentration and boosted by an externally added electron donor. In this work we wanted to study the action of oxidative enzymes (GH61) in commercial enzyme preparations during hydrolysis of an industrially relevant substrate at conditions as close as possible to a setup for bioethanol production. The substrate used was hydrothermally pretreated wheat straw at very high dry matter concentration (30% water insoluble solids, WIS), with lignin present and without the addition of electron donors as normally used in studies of oxidative enzymes. The work focused on the production of oxidized products at various process conditions, especially the impact on the β-glucosidase activity.

## Results and discussion

### Methods for the quantification of oxidized products by liquid chromatography

In previous work on elucidating the mechanism for how GH61 enzymes are acting on cellulose, various methods including e.g. MALDI-TOF, LC-MS and HPAEC have been used to detect the reaction products [10]. In most of the previous work, the focus was on detection of oxidized mono- and oligosaccharides but with less or no focus on quantifying the reaction products. In this work, the aim was to quantify the reaction products in a combined oxidative and hydrolytic breakdown of cellulose. To simplify this, the emphasis was on quantifying the final reaction products, mainly gluconic acid and cellobionic acid.

**Figure 2:**
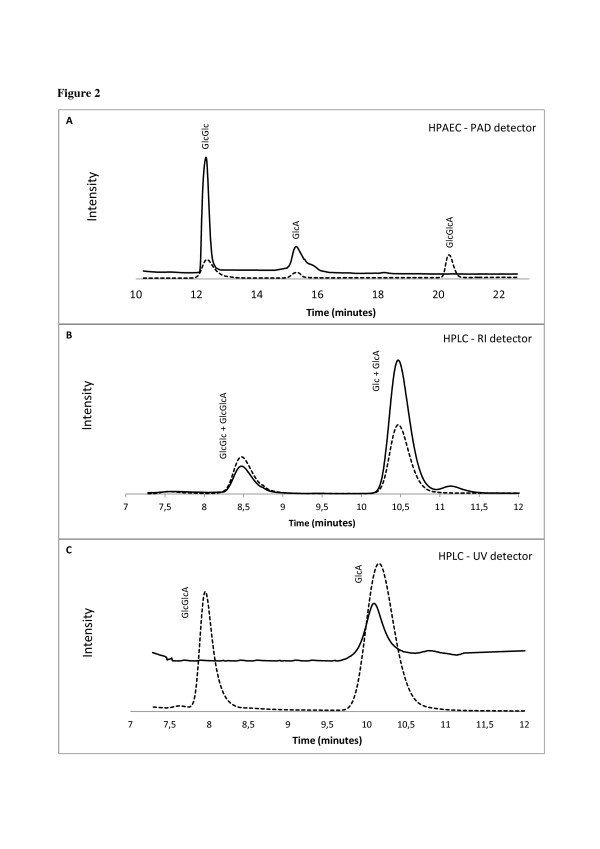
**Separation of sugars and oxidized sugars with HPAEC and HPLC systems.** Chromatograms from HPAEC with PAD ( **A**) and HPLC equipped with both RI ( **B**) and UV ( **C**) detector connected in series. In all chromatograms are shown separation of a standard sample containing cellobionic acid, cellobiose, gluconic acid and glucose (dotted line) and a sample from hydrolysis of pretreated wheat straw (solid line). The pretreated wheat straw sample was hydrolyzed for 48 hours at 50°C with Cellic CTec2 at enzyme loading of 10 mg/g DM of biomass at 30% of WIS. GlcGlcA: cellobionic acid, GlcA: gluconic acid, GlcGlc: cellobiose, Glc: glucose.

Based on previous work by Forsberg *et al.*[10], a method for separation of gluconic acid, cellobiose and cellobionic acid was investigated using a HPAEC system coupled with pulsed amperometric detection (PAD). Various modifications of the eluent composition and gradient profile were tested. Figure 2A shows that cellobiose is eluted prior to gluconic acid and cellobionic acid respectively. The method was focused mainly on the quantification of oxidized monomers and dimers as these are eventually the products that can be expected in the hydrolysate due to the presence of cellobiohydrolases and β-glucosidases.

Another HPLC method widely used for the quantification of monosaccharides and ethanol from hydrolysis and fermentation samples is based on the use of ion exclusion columns such as Bio-Rad HPX-87 H or Phenomenex Rezex ROA. The detection and quantification of oxidized products was thus tested on an HPLC system equipped with a Phenomenex Rezex ROA column (Figure 2B and 2C). Mono and oligosaccharides can only be detected on a refractive index (RI) detector, whereas oxidized sugars exhibit UV absorption and can be detected using a UV-detector at 200 nm. As shown in Figure 2B and 2C, oxidized sugars have the same retention time as their respective unoxidized native form when separated on this system. The slight shift in retention time between the RI and UV signal is due to the time delay because the detectors are connected in series. A summary of the cellobionic acid and gluconic acid elution times on both HPLC systems is given in Table 1. From a practical point of view, care should therefore be taken when analyzing hydrolysis samples on this type of system; if oxidative enzymes are present, glucose values will be overestimated due to the co-elution with gluconic acid. The glucose data have to be corrected by subtracting the gluconic acid values obtained from the UV-detector. As will be shown later, the amount of gluconic acid can in some cases make up a significant part of the glucose released from cellulose.

**Table 1:** Retention time and detection range of cellobionic acid and gluconic acid

**Aldonic acid**	**Cellobionic acid**		**Gluconic acid**	
	**HPLC-UV**	**HPAEC**	**HPLC-UV**	**HPAEC**
Retention time (minutes)	8.1	18.5	10.2	14.7
Detection range (g/l)	0.5-10	0.05-0.5	0.5-10	0.05-0.5

Both methods gave similar quantification results (in the range of 5% deviation), but the data presented in this work were reported using the HPAEC methods.

### ß-glucosidase activity in the presence of oxidized sugars

**Figure 3:**
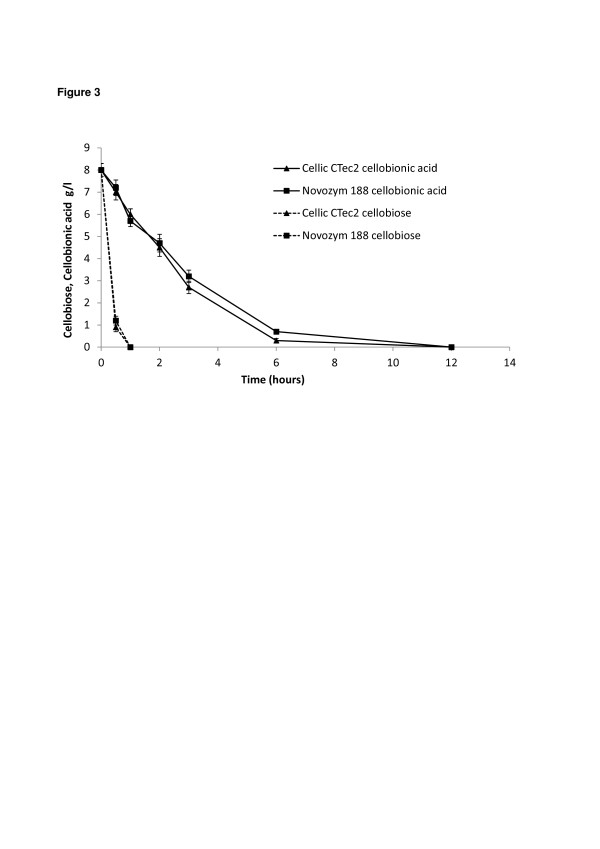
**Hydrolysis of cellobionic acid.** Hydrolysis profile of cellulose (dotted line) and cellobionic acid (solid line) using Novozym 188 (■) and Cellic CTec2 (▲). The temperature was 50°C and the enzymatic loading (calculated based on β-glucosidases activity) was similar for both preparations.

One reaction product formed after the combined action of oxidative enzymes such as GH61 and cellobiohydrolases will be cellobionic acid. Cellobiose is an inhibitor of the cellobiohydrolases, and to alleviate this product inhibition additional β-glucosidase activity is supplemented to many cellulase preparations, e.g. Celluclast. Similarly, cellobionic acid needs to be hydrolyzed to glucose and gluconic acid by β-glucosidase. The enzymatic hydrolysis of cellobionic acid by different commercial enzyme preparations (Novozym 188, Celluclast and Cellic CTec2) was measured. The Cellic CTec2 and Novozym 188 preparations were dosed based on the β-glucosidase activity to give 8 U/ml (Table 2) and were left to hydrolyze a solution containing a mix of 8 g/l of cellobiose and cellobionic acid. Under these conditions and at a temperature of 50°C, more than 6 hours was required for complete hydrolysis of cellobionic acid to glucose and gluconic acid (Figure 3). On the contrary, cellobiose was quickly hydrolyzed to glucose in less than 30 minutes. These results were similar for both enzyme preparations (Cellic CTec2 and Novozym 188). The rate of hydrolysis of cellobionic acid was also tested in a setup with the presence of Avicel cellulose, thereby mimicking the constant production of cellobiose from cellulose (data not shown). In this case, it was found that hydrolysis of cellobionic acid was even slower, 12 hours compared to 6 hours when cellobiose was only present initially. This confirms that β-glucosidase have preference for hydrolyzing the cellobiose.

**Table 2:** Properties of commercial enzyme preparations

**enzyme**	**protein content**	**FPA**	**β-glucosidase**
**preparation**	**(mg/ml)**	**(FPU/ml)**	**(U/ml)**
Celluclast 1.5 L	127	62.0	15.0
Novozym 188	220	n/a	231
Cellic CTec 2	161	120.5	2731

**Figure 4:**
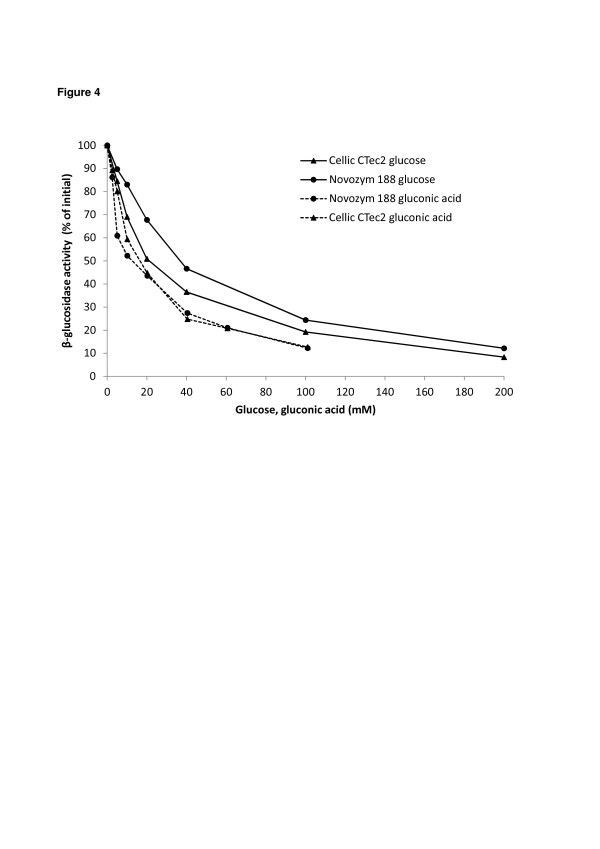
**Effect of glucose and gluconic acid on β-glucosidase activity.** Level of β-glucosidase activity in Novozym 188 (●) and Cellic CTec2 (▲) as function of gluconic acid (dotted line), and glucose (solid line) concentration. The activity was measured using p-nitrophenyl-β- D-glucopyranoside as substrate. The activity without the presence of glucose or gluconic acid was set to 100%.

Moreover, the known inhibitory effect of gluconic acid and glucose on the β-glucosidase activity [13] was tested for Cellic CTec2 and Novozym 188. The β-glucosidase activity was assayed using p-nitrophenyl-β-D-glucopyranoside as substrate and in the presence of increasing levels of gluconic acid up to 100 mM. In presence of 20 mM gluconic acid (3.92 g/l), the β-glucosidase activity was inhibited by 50% of the initial activity measured without gluconic acid (Figure 4); values close to 80% of inhibition of initial activity were achieved with 60 mM of gluconic acid for both enzyme mixtures. For comparison, a similar experiment was performed with glucose showing that at similar molar concentration, the inhibition by gluconic acid was higher than for glucose (Figure 4).

### Hydrolysis of hydrothermally pretreated wheat straw at high solids content

**Figure 5:**
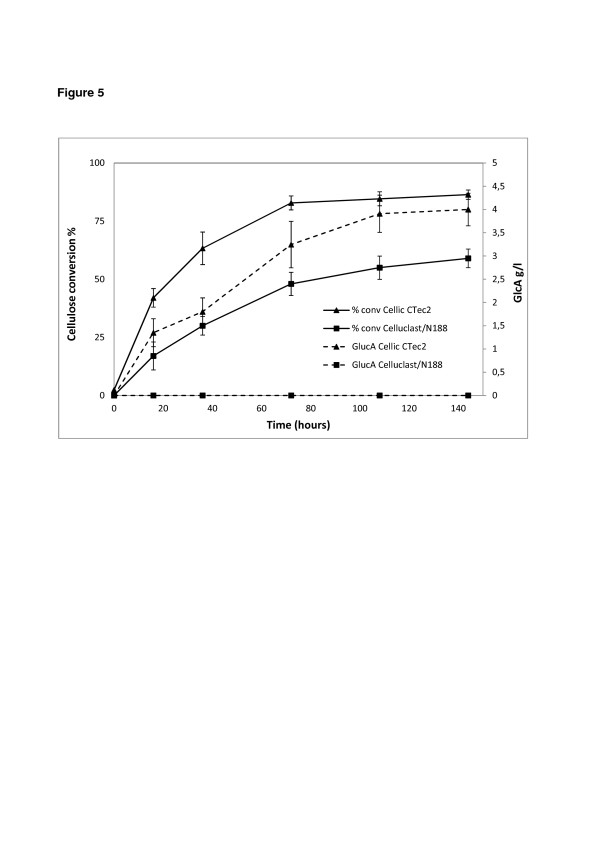
**Cellulose conversion and gluconic acid production during hydrolysis of pretreated wheat straw using Cellic CTec2 and Celluclast-Novozym 188.** Cellulose conversion (solid line) and gluconic acid concentration (dotted line) during hydrolysis of pretreated wheat straw using Cellic CTec2 (▲) and Celluclast-Novozym 188 (■). The hydrolysis was performed using an enzyme loading of 7.5 FPU/g DM at 50°C and a solids loading of 30% WIS.

Hydrothermally pretreated wheat straw from a pilot scale facility was used for investigating the formation of oxidized sugars during deconstruction of a lignocellulosic substrate at high solids content (30% WIS), which is relevant for industrial conversion. The results in Figure 5 show the production of gluconic acid along with the degree of conversion during the time course of the hydrolysis using Cellic CTec2 and Celluclast supplemented with Novozym 188. Both enzyme preparations were dosed to give a filter paper activity of 7.5 FPU/g DM. After 144 hours, Cellic CTec 2 yielded 85% cellulose conversion and 4.1 g/l of gluconic acid. By comparison the Celluclast-Novozym 188 mixture resulted in 60% conversion and no gluconic acid was detected with this enzyme. In both experiments, no cellobionic acid was detected in a quantifiable amount during the hydrolysis, although traces were observed at high values of cellulose conversion or at long hydrolysis times using Cellic CTec2. This shows that under the given conditions the level of β-glucosidase activity is enough to ensure conversion of cellobionic acid although the enzymes also have to hydrolyze cellobiose. The results clearly show that the latest cellulase preparations are supplemented with an oxidative enzyme, most likely from the family GH61 [16] and that in earlier preparations these enzymes are deficient or not active. From the experiment it was found that under conditions close to industrial settings, approximately 4.1% of the glucose released ended up as gluconic acid. Since work showing the oxidative effect of the CDH enzymes during lignocellulosic hydrolysis was recently published [19], we tested the possible presence of such proteins in the enzymatic preparations (data not shown). The CDH enzyme is capable of oxidizing cellobiose to cellobionic acid, but no gluconic acid was found when a solution containing only cellobiose was hydrolyzed with Cellic CTec2. The oxidation observed with Cellic CTec2 seems therefore not to be due (or partly due) to CDH enzymes. The proposed main activity of GH61 enzymes is the oxidative cleavage of endo-glycosidic bonds in cellulose, thus the amount of gluconic acid is at least equal to the number of new entry sites created in the cellulose backbone due to the action of GH61. Considering that 4.1% of the glucose released from the cellulose ends up as gluconic acid and that cellobiohydrolase activity also contributes to the yield of glucose, then a considerable number of the endo-glycosidic bonds must have been oxidatively cleaved by GH61 and not by endoglucanases. Thus, the inclusion of GH61 enzymes in the latest generations of cellulase preparations has a large impact upon the number of entry sites for hydrolytic enzymes. Further exploration of the dynamics and interactions between cellulose oxidases and hydrolases may improve the performance of the enzyme preparations even more. From an industrial point of view it should also be considered if the level of gluconic acid produced is acceptable since it might not be metabolized into the desired product of fermentation.

### Gluconic acid production: Effect of temperature

**Figure 6:**
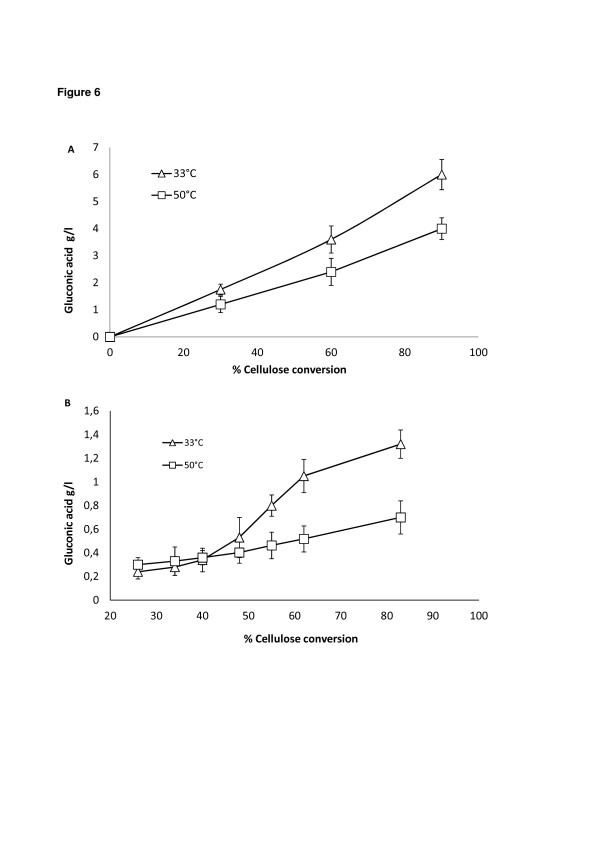
**Gluconic acid production as function of cellulose conversion.** Gluconic acid concentration as function of cellulose conversion using Cellic CTec2 at 33°C (Δ) and 50°C (□) with pretreated wheat straw ( **A**) and filter paper ( **B**) as substrate. The enzyme loading was 7.5 FPU/g DM and the solids concentration was 30% WIS of wheat straw and 5% WIS of filter paper. In the case of filter paper 20 mM ascorbic acid was added as external electron donor.

The formation of gluconic acid was investigated at two different temperatures: 50°C and 33°C corresponding to typical temperatures used for SHF and SSF, respectively. Pretreated wheat straw and filter paper were used as substrates. In the filter paper experiments ascorbic acid was added as a reducing agent needed for the activity of GH61 enzymes [5-7,9,10]. There is a clear difference in gluconic acid production at the two temperatures (Figure 6), and less gluconic acid was produced from both substrates when performing hydrolysis at 50°C compared to 33°C. For pretreated wheat straw the final gluconic acid concentration after 108 hours was found to be 3.9 g/l and 6.1 g/l using 50°C and 33°C, respectively. For filter paper, at 83% of glucose conversion, the concentration of gluconic acid was 1.36 g/l and 0.7 g/l using 33°C and 50°C, respectively; the amount of gluconic acid was 0.1 g/l when no ascorbic acid was added to Cellic CTec2 and the hydrolysis was run at 50°C with same conversion of glucose of the latter. Normalizing the data by weight of cellulose in the two substrates, it can be calculated that from 100 grams of cellulose, 2.1 and 2.5 grams of gluconic acid was produced from filter paper and pretreated wheat straw at 50°C respectively, and at 33°C the yields were 3.4 and 3.9 grams of gluconic acid by the same materials, respectively. Thus the yield of gluconic acid from filter paper in the presence of ascorbic acid was almost the same as that of wheat straw without reducing agents being added. We suggest that in the case of lignocellulosic materials, lignin can supply the electrons needed for the oxidation step by behaving as a reducing agent. This is well in accordance with previous observations suggesting that lignin in general is part of redox cycles [20]. The difference observed between 33° and 50°C may indicate that GH61 enzyme(s) are not stable at the higher temperature or that the activity of the cellobiohydrolases and endoglucanases decrease more than the activity of the GH61 enzymes when the temperature is lowered. The latter mechanism is more likely as the rate of gluconic acid formation is proportional to the rate of glucose formation throughout the hydrolysis (Figure 6). From an industrial point of view it is interesting to note that operating the high solids hydrolysis of lignocellulosic material following an SHF (50°C) configuration leads to less gluconic acid produced (2.8% of glucose released) compared to the SSF (33°C) giving 4.1% of glucose released. Therefore, by choosing a SHF process, less glucose will end up as gluconic acid which cannot be fermented to ethanol by e.g. *S. cerevisiae* and there will be a lower level of inhibition of the β-glucosidase enzymes by gluconic acid. The choice of operating conditions is therefore not only a matter of optimizing the performance with respect to the rate of hydrolysis and overall process time but also by-products yield.

## Conclusions

Oxidative enzymes such as GH61 increase the overall yield of hydrolytic enzyme preparations. In this work we found that under commercially relevant conditions, around 4.1% of the glycosidic bonds in cellulose were oxidatively cleaved by presumably GH61 enzymes, which provides new entry sites for the hydrolytic enzymes if aggregate or crystalline cellulose is the only substrate. Then, accounting for other oxidized forms of glucose such as 4-ketoaldoses resulting from C4 oxidation (not quantified in this work) [21], then the final contribution to the total pool of entry sites created by GH61s could be even higher. Moreover, the results show that the β-glucosidase enzymes contained in the Cellic Ctec2 mixture are able to hydrolyze cellobionic acid but at a rate much slower compared to cellobiose. For such enzyme preparations containing GH61 enzymes, the classical model of action of cellulolytic enzymes should be modified to include the hydrolysis of cellobionic acid by β-glucosidases. We also found that in substrates where lignin is present, no added reducing agent is needed for GH61 to function. This indicates a link between the oxidative breakdown of cellulose and redox cycles in lignin through electron transport mechanisms. Moreover, the data presented in this work revealed that a significant amount of glucose was oxidized into non-fermentable gluconic acid and its effect as an inhibitor of the β-glucosidases was documented. Depending on temperature, at least 4–6 g/l of gluconic acid was produced (higher concentration at lower temperature), and this amount was sufficient to result in 50% inhibition of the β-glucosidase activity in the enzyme preparations. Furthermore, this work will be relevant for the characterization of the metabolic effects of gluconic acid at such concentrations on the glucose uptake and fermentation of the yeast *S. cerevisiae*.

## Methods

### Chemical oxidation of cellobiose

Cellobiose (Sigma Aldrich, USA) was oxidized using a mild oxidation method that has been shown to selectively oxidize the hemiacetal carbon (C1) of carbohydrates to generate aldonic acids. The experiment was performed following the method described by Forsberg *et al.*[10]. The quantification of cellobionic acid obtained after the oxidation was done indirectly by measuring the quantity of gluconic acid and glucose generated by β-glucosidase hydrolysis from *Aspergillus niger* (Novozym 188, Novozymes A/S, Bagsværd, Denmark).

### Wheat straw pretreatment and compositional analysis

Wheat straw (*Triticum aestivum* L.) was pretreated at the Inbicon A/S pilot plant in Skærberg, Denmark, with an average residence time of 18.5 min in a hydrothermal reactor with a temperature of 195°C. A washing and pressing step was applied prior the enzymatic hydrolysis to eliminate the soluble molecules hydrolyzed and generated during the pretreatment e.g. pentose sugars as xylose mainly and toxicants as furfural, HMF and acetic acid; a final dry matter content of 32,5% was achieved [22]. No chemicals were added during the pretreatment. The composition of the solid material was analysed by strong acid hydrolysis using a modified version of the TAPPI standard procedure [23], the modification being that the standard curve samples were treated similarly to the samples to correct for sugar degradation. Before analysis the material was washed with water to remove soluble sugars by repeated centrifugation and suspending in deionized water. The solids were then dried at 60°C over night. The monosaccharides D-glucose, D-xylose, L-arabinose, D-mannose and D-galactose (standards from Sigma Aldrich, USA) were measured on a Dionex ICS5000-system equipped with a CarboPac-PA1 column and using PAD-detection (Dionex, Sunnyvale, CA, USA). The flow rate was 1.0 ml/min and the eluent was MilliQ-water for 35 min followed by an increase to 200 mM NaOH for 10 min and a final equilibration with MilliQ-water for 10 min. To improve detection post-column mixing with 200 mM NaOH at 0.5 ml/min was used. The chemical composition of filter paper and wheat straw is shown in Table 3.

**Table 3:** Chemical composition of pre-treated wheat straw and filter paper analyzed using the two-stage acid hydrolysis method

**Structural**	**Pre-treated Wheat**	**Filter paper**
**component**	**straw % DM**	**% DM**
**Cellulose**		
Glucan	**53.69**	**84.00**
**Hemicellulose**	**4.18**	**4.28**
Xylan	3.53	1.44
Mannan	0.49	2.02
Arabinan	0.06	0.33
Galactan	0.10	0.49
**Klason lignin**	**34.1**	**0**
**Ash**	**6.07**	**0.74**
**Total**	**98.04**	**94.42**

### Enzymes mixture

Cellulolytic enzyme complex from *Trichoderma reesei* in the form of a commercial mixture (Celluclast 1.5 L), cellulases with addition of GH61from a genetically modified strain of *T. reesei* (Cellic CTec2), and β-glucosidase from *Aspergillus niger* (Novozym 188), were all obtained from Novozymes A/S, Bagsværd, Denmark. The filter paper activity was determined according to Ghose *et al.*[24]; the β-glucosidase activity was measured using 5 mM p-nitrophenyl-β-D-glucopyranoside (Sigma Aldrich) as substrate [25]. Protein content was measured using the Ninhydrin assay with BSA as protein standard [26]. A summary of the results is shown in Table 2.

### Hydrolysis of wheat straw

Hydrolysis of pretreated wheat straw (120 grams) was performed in 500 ml blue cap bottles. Water was added to adjust the solid loading to 30% WIS. The pH was adjusted to 5.0 by addition of 1 ml of 13.5 M aqueous NaOH. The material was mixed using a roller bottle reactor system [27]. The enzyme loading was 7.5 FPU/g DM (10 mg/g DM) of Cellic CTec2 preparation, and Celluclast mixed with Novozym 188 (with a ratio of 5:1, final enzymatic loading 17.7 mg/g DM); the temperature was set at 33°C and 50°C. The experiment set at 33°C was designed to emulate the temperature during a SSF process, and a pre-hydrolysis step at 50°C was applied until the achievement of liquefaction (16 hours), after which the material was cooled to 33°C and hydrolyzed for 128 hours. The experiment at 50°C was hydrolyzed for 96 hours. The samples were taken directly from the reactor during hydrolysis, and then were prepared according to Kristensen *et al.*[28] due to the higher density of the hydrolysate (final density equal to 1.12) AT 30% of WIS with respect to water. The analytes were quantified in weight/weight (g/Kg) unit of measure, then converted to weight/volume units (g/L) multiplying by the density factor measured at every time point. A boiling step of 10 minutes at 105°C was applied prior to weighing the material and diluted with the appropriate amount of milli-Q water to the final concentration of carbohydrates, and finally centrifuged.

### Hydrolysis of filter paper

The hydrolysis of filter paper (2.5 grams) was done in 100 ml blue cap bottles using an incubator with orbital shaker set at 150 rpm, the temperature was 50°C as well as 33°C and hydrolysis length of 96 hours. The solid loading was 5% (w/w) and the experiment was done with and without ascorbate (20 mM) in 50 mM Na-citrate buffer at pH 4.8. The enzymatic loading was 4 FPU/g of filter paper for both enzymatic preparations: Cellic CTec2 (5.3 mg/g of biomass), and Celluclast plus Novozym 188 (mixed with a ratio of 5:1, final enzymatic loading 9.44 mg/g DM).

### Hydrolysis of cellobiose and cellobionic acid

The hydrolysis of cellobiose and cellobionic acid (synthesized in this work) was done using a solution containing 8 g/l of both compounds. The enzymes (Cellic CTec2 and Novozym188) were dosed based on β-glucosidase activity to give 8 U/ml. The reaction was performed at 33°C in Eppendorf tubes with constant shaking. The samples were boiled for 10 minutes to stop the reaction, filtered through a 0.45 μm pore size filter, then analyzed with HPAEC chromatography (ICS5000 equipped with CarboPac PA1 column, Dionex, CA, USA). The inhibition of the β-glucosidase activity was assayed including different concentration of inhibitors (glucose and gluconic acid, from 2 to 200 mM) in a solution containing 5 mM p-nitrophenyl-β-D-glucopyranoside as substrate. The quantity of p-nitrophenol released in presence or absence of inhibitors was measured by UV-absorbance (405 nm) in alkaline environment upon inhibition of the enzymes; the activity assays was done at 50°C for 15 minutes in 50 mM Na-citrate buffer at pH 4.8.

### Analysis of carbohydrates and oxidized products

The quantification of D-glucose, D-cellobiose, gluconic acid (Sigma Aldrich, USA) and cellobionic acid (synthesized in this work), was done on two different HPLC instruments: i) UltiMate 3000 HPLC (Dionex, Germering, Germany) equipped with refractive index detector (Shodex, Japan) and UV detector at 200 nm (Dionex). The separation was performed in a Phenomenex Rezex ROA column at 80°C with 5 mM H_2_SO_4_ as eluent at a flow rate of 0.6 ml/min. The results were analyzed using the Chromeleon software program (Dionex). ii) ICS5000 HPAEC coupled with PAD (Dionex, Sunnyvale, CA, USA). The separation was performed using a Dionex CarboPac PA1 analytical column. In this system only D-cellobiose, gluconic acid and cellobionic acid were separated and quantified. The column was operated at a flow of 1 ml/min and maintained at 30°C. The eluent was 0.1 M NaOH, and a solution of 0.2 M of NaOH was applied post column prior to the detector at 0.5 ml/min. Several programs with a stepwise linear gradient with increasing concentration of sodium acetate were applied. A well defined peak separation was obtained when applying the following elution gradient: 0.1 M NaOH for 5 minutes, then a linear increase from 0.1 M NaOH to 0.1 M NaOH with 0.3NaOAc in 35 minutes, then to 0.1 M NaOH/1 M NaOAc in 5 minutes. The column was reconditioned with 0.1 M NaOH for 5 min before next sample.

## Abbreviations

CDH, Cellulose dehydrogenase; DM, Dry mater; Glc, Glucose; GlcA, Gluconic acid; GlcGlc, Cellobiose; GlcGlcA, Cellobionic acid; HPAEC-PAD, High performance anion exchange chromatography/pulsed amperometric detector; HPLC-PDA, High performance liquid chromatography/photodiode array detector; LC-MS, Liquid chromatography-mass spectrometry; MALDI-TOF, Matrix-assisted laser desorption/ionization time of flight mass spectrometry; NZ188: Novozym 188; SHF, Separate hydrolysis and fermentation; SSF, Simultaneous saccharification and fermentation; WIS, Water insoluble solids.

## Competing interests

The authors declare that they have no competing interests.

## Authors’ contributions

All authors contributed jointly to all the aspects of the work reported in the manuscript. DC and HJ planned the experimental work. DC carried out the experimental work. DC and CCH contributed to the interpretation of the results and writing of the manuscript. CF and HJ contributed to the interpretation, supervision, critical review and writing of the manuscript. All authors read and approved the final manuscript.
